# Characterization of the First Complete Plastome of *Zingiber emeiense*: Insights Into Its Phylogenetic Position

**DOI:** 10.1002/ece3.74465

**Published:** 2026-10-25

**Authors:** Hualan Chen, Zhaofei Wang, Jinyong Ni, Qinxi Feng, Yue Liu, Lihui Jiang, Chao Song, Yanlin Zhu, Menghan Zhang, Qin Xie, Maoqin Xia, Honglei Li

**Affiliations:** ^1^ Chongqing Engineering Research Center for Horticultural Plant, College of Smart Agriculture Chongqing University of Arts and Sciences Chongqing China; ^2^ Chongqing Key Laboratory for Germplasm Innovation of Special Aromatic Spice Plants, College of Smart, Agriculture Chongqing University of Arts and Sciences Chongqing China

**Keywords:** phylogenomics, plastome, *Zingiber*, *Zingiber emeiense*

## Abstract

*Zingiber emeiense* Z. Y. Zhu is an endemic medicinal species restricted to Mount Emei in southwestern China. However, the lack of genomic resources has led to its frequent misclassification and has impeded evolutionary studies. Here, we assembled and characterized the first two complete chloroplast genomes (plastomes) of *Z. emeiense*. The two plastomes were identical in sequence and structure, each with a total length of 163,865 bp and exhibiting a typical quadripartite structure. Each plastome comprised a large single‐copy (LSC) region of 88,191 bp, a small single‐copy (SSC) region of 15,750 bp, and a pair of inverted repeats (IR) of 29,962 bp each. Each genome encoded 134 functional genes, including 86 protein‐coding genes, 40 tRNA genes, and eight rRNA genes, with an overall GC content of 36.04%. Comparative plastome analyses revealed that IR boundary variation and repeat sequence differences were major sources of genome size variation within *Zingiber*. Seven hypervariable regions (*psb*M, *trn*T‐UGU, *ycf*1, *pet*A‐*psb*J, *ndh*F, *psa*C‐*ndh*E, and *rps*19) were identified as potential molecular markers. Likelihood‐based selection analyses revealed that plastid gene evolution was primarily driven by purifying selection, but ten genes showed signatures of positive selection; *ycf*1, *pet*D, and *rpl*2 exhibited lineage‐specific positive selection on the *Z. emeiense* clade. Phylogenomic reconstruction based on 70 complete plastomes placed *Z. emeiense* within sect. *Cryptanthium*, where it was strongly supported as a sister clade to *Zingiber smilesianum* and *Zingiber teres*. This study provides essential genomic resources for conservation and taxonomic studies of this endemic species.

## Graphical abstract

This study assembled the first complete chloroplast genome of the endemic medicinal plant *Zingiber emeiense*, revealing its genomic structure and evolutionary features as well as clarifying its phylogenetic placement. It further provides valuable genomic resources for the conservation and taxonomic research of this endemic species.

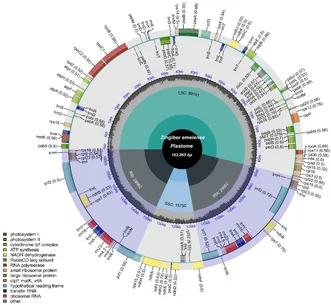

## 1 Introduction

*Zingiber* Mill. is a perennial herbaceous genus of the family Zingiberaceae, including 100–150 species (Ding et al. 2021, 2023; Jayakrishnan et al. 2021; Lý et al. 2021). Species of this genus are mainly distributed from tropical to warm temperate regions of Asia and are valued for their economic and ornamental uses (Zhu 1984). Within this genus, *Zingiber emeiense* Z. Y. Zhu is a species endemic to Mount Emei in Sichuan Province, China (Zhu 1984; Bai 2016; Xiao 2016). This species has attracted growing research interest due to its pronounced pharmacological potential. Its red rhizome and infructescence possess multiple bioactivities, including hematopoietic, cardiotonic, and anxiolytic effects, and show clinical efficacy in the treatment of palpitations, cardiac fatigue, and cough (Zhu 1984; Bai 2016; Deng et al. 2022).

Beyond its medicinal significance, *Z. emeiense* displays unique ecological and morphological traits. It occupies a specialized mid‐elevation habitat (*c*. 900 m a.s.l.) in moist, shaded broad‐leaved forests, a distribution distinct from the lowland rainforest typical of most congeners (Fang 1982; Bai et al. 2018). Morphologically, *Z. emeiense* is characterized by an ovoid‐elliptical spike bearing 13–28 flowers, distinctive purple lateral stamens with white stripes, and most notably, an elongate, slender, creeping rhizome reaching up to 1.5 m in length (Bai et al. 2018) (Figure 1). This rhizome architecture is rare within the genus and shared by only five other species, namely *Zingiber leucochilum* L.Bai, Škorničk. & N.H.Xia, *Zingiber leptorrhizum* D.Fang, *Zingiber pauciflorum* L.Bai, Škorničk., D.Z.Li & N.H.Xia, *Zingiber smilesianum* Craib, and *Zingiber yunnanense* S.Q.Tong & X.Z.Liu (Fang 1982; Xiao 2016; Bai et al. 2018).

**FIGURE 1:**
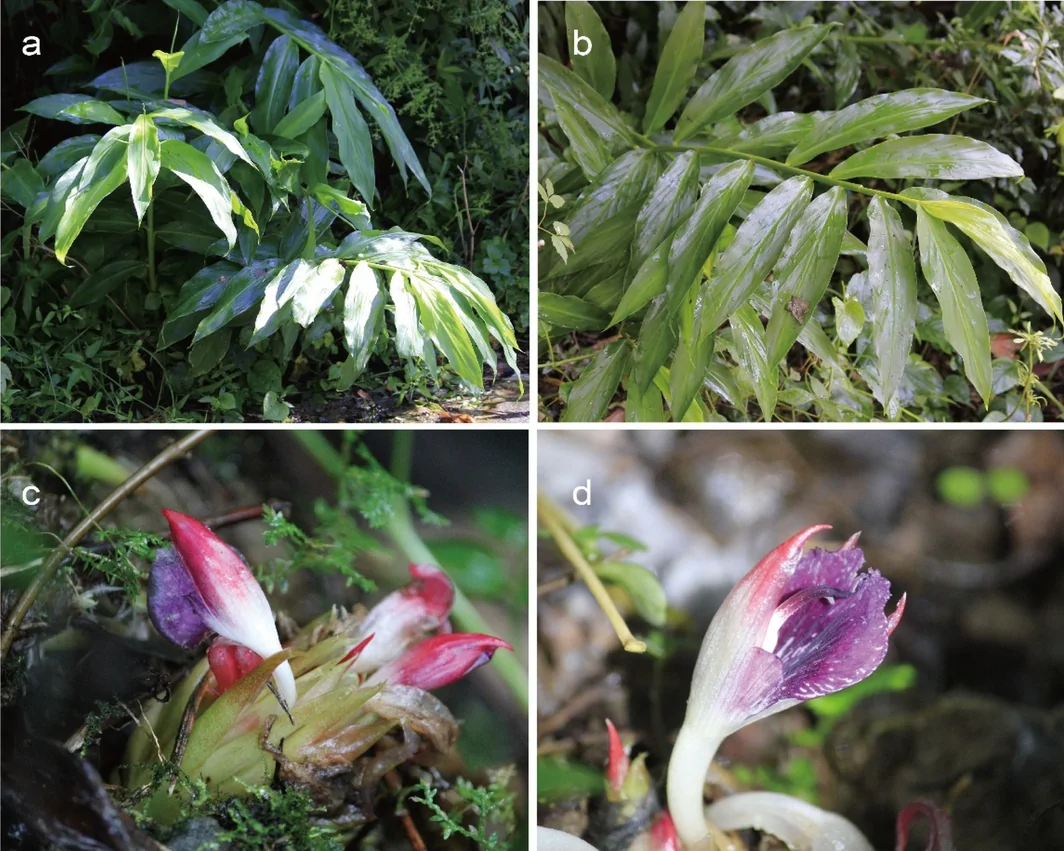
Morphological characteristics of *Zingiber emeiense*. (a) Plants of *Z. emeiense* in its natural habitat; (b) leaf morphology; (c) inflorescence; (d) floral morphology. Photographed by Hualan Chen.

Traditionally, the genus *Zingiber* has been classified into four sections based on the position of the inflorescence: sect. *Zingiber*, characterized by a basal inflorescence with a long, erect peduncle; sect. *Dymczewiczia*, bearing a terminal inflorescence; sect. *Pleuranthesis*, producing an inflorescence emerging laterally from the leaf sheath; and sect. *Cryptanthium*, possessing a basal inflorescence with a prostrate peduncle (Horaninow 1862; Bentham and Hooker 1883; Leong‐Skornickova et al. 2015; Jayakrishnan et al. 2021). *Zingiber emeiense* displays a basal, prostrate‐peduncled inflorescence, a diagnostic trait of section *Cryptanthium* (Zhu 1984; Bai 2016; Xiao 2016). Notably, *Z. emeiense* has been misidentified as a synonym of *Zingiber striolatum* Diels in Flora of China (FOC) (Wu and Larsen 2000). However, these two taxa are morphologically distinct and can be differentiated by key traits. For example, *Z. emeiense* possesses a slender, creeping rhizome, longer peduncle, more loosely arranged bracts, and flowers that extend more conspicuously beyond the bracts, while *Z. striolatum* has a stout, compact rhizome, and a more compact inflorescence (Zhu 1984; Bai 2016). The two conflicting morphology‐based treatments of *Z. emeiense*—its recognition as a distinct species versus its synonymization under *Z. striolatum* in Flora of China—remain untested by independent data. Resolving this controversy requires molecular evidence, as species delimitation in *Zingiber* relies largely on floral and rhizome characters, which overlap and vary continuously among congeners, and are frequently obscured in herbarium specimens (Zhu 1984; Bai 2016). Complete plastomes, which provide genome‐scale characters independent of morphology and have proved informative in resolving phylogenetic relationships within *Zingiber* (Jiang et al. 2023; Xia et al. 2024), offer a robust molecular basis for evaluating the taxonomic status of *Z. emeiense*. In addition, as an endemic species with unique morphological adaptations and significant pharmacological value, *Z. emeiense* served as a valuable genetic resource for systematics, conservation, and sustainable utilization of *Zingiber*. Yet targeted studies focusing on *Z. emeiense* remain extremely scarce, which greatly impedes its taxonomic resolution, conservation evaluation, and rational exploitation.

Chloroplasts are essential organelles of green plants, acting as the main sites of photosynthesis and containing their own independent genomes (Wicke et al. 2011; Daniell et al. 2016). Chloroplast genomes (plastomes) exhibit a typical circular quadripartite structure, comprising a pair of inverted repeats (IRs) that are separated by a large single‐copy (LSC) region and a small single‐copy (SSC) region. Plastomes of most angiosperms range from 120 to 160 kb in size and encode about 100–120 functional genes (Li et al. 2019). Compared to mitochondrial and nuclear genomes, plastomes possess unique advantages, including high genetic conservation, a slow evolutionary rate, predominantly maternal inheritance, and abundant mutation sites (Tsunewaki and Ogihara 1983; Jiang et al. 2023). These characteristics render plastomes ideal for species identification and phylogenetic inference, especially for morphologically similar species that are difficult to distinguish using traditional taxonomic approaches (Xia et al. 2024). Complete plastomes have been widely employed to resolve phylogenetic relationships within *Zingiber* (Jiang et al. 2023). Despite their utility, publicly available complete plastomes currently represent less than 50% of the genus (Wang et al. 2026), highlighting a critical gap in genomic resources for understanding species delimitation and evolutionary history in *Zingiber*.

Despite its endemic status and pharmacological value, no plastome data have been reported for *Z. emeiense*, leaving its phylogenetic placement within the genus unresolved and impeding conservation work. In this study, we generated complete plastome sequences from two individuals of *Z. emeiense* and analyzed them together with available plastomes of the genus, with the aims to (1) characterize the structural features, gene content, and repetitive elements of the *Z. emeiense* plastome; (2) identify hypervariable regions and evaluate selective pressures across *Zingiber* plastomes; and (3) resolve the phylogenetic position of *Z. emeiense* within the genus. Our research is expected to clarify the phylogenetic relationship between *Z. emeiense* and *Z. striolatum*, and the resulting genomic resources will support future classification and conservation studies of *Zingiber*.

## 2 Materials and Methods

### 2.1 Sample Collection, DNA Extraction, and Sequencing

Fresh leaves of two samples of *Z. emeiense* (emei1 and emei2) were collected from Mount Emei, Sichuan Province, China at coordinates 103.221321° E, 29.354896° N and 103.353185° E, 29.61233° N respectively. The voucher specimens have been deposited at Chongqing University of Arts and Sciences under the herbarium numbers XMQ2025067‐1 and XMQ2025070‐1. They were identified by Maoqin Xia (Contact: xiamq@cqwu.edu.cn). Genomic DNA was extracted from approximately 50 mg of fresh leaf tissue using the Plant Genomic DNA Extraction Kit (Tiangen, Beijing, China). After passing quality control, the DNA was loaded onto a Covaris E220 system (Covaris, Brighton, UK) for ultrasonication‐based fragmentation, producing fragments of 300–500 bp. The fragmented DNA was subjected to a series of enzymatic reactions, including end repair, A‐tailing, and ligation with indexed adapters. Following PCR amplification, the products were circularized to create single‐stranded circular (ssCir) libraries. Rolling circle amplification (RCA) was then applied to these libraries to form DNA nanoballs (DNBs), which were finally sequenced on the MGI‐DNBSEQ platform (Shenzhen, China) with 150 bp paired‐end chemistry (Wang et al. 2026).

### 2.2 Plastome Assembly and Annotation

Raw reads were processed with Fastp v1.0.1 to discard those containing > 10% ambiguous nucleotides (N) or > 50% of bases with Phred quality scores (Q) < 5 (Chen et al. 2018). The clean reads, which achieved Q20 and Q30 scores of 99% and 96%, respectively, were subsequently used for *de novo* assembly of the plastome with GetOrganelle v1.7.7.1 (Jin et al. 2020). We performed 15 rounds of extension iterations (‐R 15) to obtain a complete plastome. K‐mer parameters (‐k) were set to 21, 45, 65, 85, and 105. The assembled plastomes were annotated in Geneious Prime 2025 (Kearse et al. 2012) with the plastome of *Z. striolatum* (OM868073) as the reference. Exon‐intron boundaries and start/stop codon positions of all genes were manually checked. Finally, the plastome of *Z. emeiense* was visualized using CPGView (Liu et al. 2023).

### 2.3 Relative Codon Usage

Relative synonymous codon usage (RSCU) is a key metric for quantifying codon usage bias. We used CodonW v1.4.2 (http://codonw.sourceforge.net) to analyze the codon usage bias, effective number of codons (ENC), and GC content at different codon positions of the five *Zingiber* plastomes, i.e., *Z. emeiense*, *Zingiber atroporphyreum* Škorničk. & Q.B.Nguyen, *Zingiber ellipticum* (S. Q. Tong & Y. M. Xia) Q. G. Wu & T. L. Wu, *Zingiber officinale* Roscoe, and *Zingiber mioga* (Thunb.) (Table 1). Each of these species represents one of the four sections of the genus recognized on the basis of inflorescence morphology and position: *Z. officinale* (sect. *Zingiber*), *Z. mioga* (sect. *Cryptanthium*), *Z. ellipticum* (sect. *Pleuranthesis*), and *Z. atroporphyreum* (sect. *Dymczewiczia*). A RSCU value of 1 indicates no bias among synonymous codons, while RSCU > 1 denotes preferentially used codons and RSCU < 1 indicates low‐frequency codons (Li et al. 2025).

**TABLE 1:** Plastome feature comparisons of *Zingiber emeiense* with other related species.

Species	*Z. atroporphyreum*	*Z. ellipticum*	*Z. officinale*	*Z. emeiense*	*Z. mioga*
Accession number	N_001486761	OP869976	OR337873	PZ174020/PZ174021	OR337872
Total length (bp)	163,880	163,455	162,921	163,865	163,511
Total% GC	36.09	36.16	36.08	36.04	36.04
LSC length (bp)	88,460	87,946	87,486	88,191	88,026
LSC%GC	33.84	33.89	33.81	33.84	33.79
SSC length (bp)	15,926	15,771	15,577	15,750	15,871
SSC%GC	29.54	29.78	29.74	29.57	29.39
IR length (bp)	29,747	29,869	29,929	29,962	29,807
IR%GC	41.21	41.19	41.04	40.98	41.13
Protein‐coding gene	86	86	86	86	86
tRNAs	38	38	38	40	38
rRNAs	8	8	8	8	8

*Note:* The plastome of *Z. atroporphyreum* was obtained from the China National GeneBank (CNGB); all other sequences were obtained from GenBank. The plastomes of the two *Z. emeiense* individuals are completely identical in sequence and structure, with the same genomic features shown in the table.

### 2.4 Comparative Plastome Analyses

We performed a comparative analysis of the plastomes of *Z. emeiense* and four representative species from the four sections of *Zingiber*, i.e., *Z. atroporphyreum*, *Z. ellipticum*, *Z. officinale* , and *Z. mioga* (see above). The contraction and expansion patterns at the LSC/IRb/SSC/IRa boundaries of these plastomes were examined using the online tool CPJSdraw v1.0.0 (Li et al. 2023).

Dispersed repetitive sequences in the plastomes of *Z. emeiense* and representative species from the four sections of the genus *Zingiber* were identified using the online software REPuter (https://bibiserv.cebitec.uni‐bielefeld.de/reputer) (Kurtz et al. 2001). We identified repeats of four types, including forward (F), palindromic (P), reverse (R), and complement (C). The analysis was performed for repeats ranging in length from 30 to 500 bp, and the Hamming distance was set as 3. Redundant, nested, and overlapping repeats in the raw REPuter output were removed, so that each unique repeat locus was counted only once. Simple sequence repeats (SSRs) were detected with MISA (https://webblast.ipk‐gatersleben.de/misa) (Beier et al. 2017) using minimum repeat thresholds of 8, 5, 4, 3, 3, and 3 for mono‐, di‐, tri‐, tetra‐, penta‐, and hexanucleotide repeats, respectively.

Sequence divergence among the five *Zingiber* species (*Z. atroporphyreum*, *Z. ellipticum*, *Z. officinale* , *Z. emeiense*, and *Z. mioga* ) was analyzed using mVISTA (https://genome.lbl.gov/vista/mvista/submit.shtml) (Frazer et al. 2004) under the Shuffle‐LAGAN mode, and with *Z. atroporphyreum* as the reference. To identify highly divergent chloroplast genomic regions among them, we first aligned their complete plastome sequences using the MAFFT plugin in Geneious Prime 2025. Then, nucleotide diversity (*π*) was calculated using DnaSP v6.10 with a sliding window of 600 bp and a step size of 200 bp (Rozas et al. 2017).

### 2.5 Analysis of Selective Pressure

Selection pressure acting on plastid protein‐coding genes was investigated using likelihood‐based codon‐substitution models implemented in HyPhy v2.5.101 (Kosakovsky Pond et al. 2020). Sequences of 74 protein‐coding genes (CDS) shared among 32 *Zingiber* species (one representative accession per species, including *Z. emeiense*; Table S1) were extracted from the annotated plastomes as input data. For each gene, terminal stop codons were removed. Codon‐aware multiple sequence alignments were constructed using MAFFT v7.525 via amino acid alignment and subsequent back‐translation to codons (Katoh and Standley 2013). The phylogenetic framework for all selection tests was derived by pruning the whole‐plastome maximum‐likelihood phylogeny (see below) to the corresponding taxa, and branch lengths were re‐estimated under each codon model. Four complementary tests were conducted for every gene: (1) BUSTED, which tests for gene‐wide episodic diversifying selection (Murrell et al. 2015); (2) aBSREL, which infers the optimal number of *ω* (dN/dS) rate classes per branch and tests branch‐specific positive selection with built‐in multiple‐testing correction (Smith et al. 2015); (3) MEME, which detects episodic diversifying selection at individual codon sites (Murrell et al. 2012); and (4) FEL, which tests for pervasive selection at individual sites (Kosakovsky Pond and Frost 2005). Statistical significance was set at *p* < 0.05, and BUSTED *p* values across genes were further adjusted using the Benjamini–Hochberg false discovery rate procedure (Benjamini and Hochberg 1995).

### 2.6 Phylogenetic Analysis

To resolve the phylogenetic position of *Z. emeiense* within *Zingiber*, we performed phylogenetic inference using 70 complete plastomes representing 54 species (Table S1). All plastomes were aligned with MAFFT v7 and concatenated into a super‐matrix. Phylogenetic reconstruction was conducted using maximum likelihood (ML). The ML analysis was carried out in IQ‐TREE v1.6.12, where the optimal substitution model and partitioning scheme were simultaneously determined under the Bayesian information criterion (BIC) using ModelFinder (Zhou et al. 2006; Kalyaanamoorthy et al. 2017). The ML tree was reconstructed under the GTR + F + I + R3 model, and branch support was assessed with 1000 ultrafast bootstrap replicates. The resulting trees were visualized using iTOL v6 (https://itol.embl.de/) (Letunic and Bork 2021).

## 3 Results

### 3.1 Structural Characteristics of the Plastome

The two plastomes of *Z. emeiense* were identical in sequence and exhibited a typical quadripartite structure. The complete genome of each individual was 163,865 bp in length, with the LSC and SSC regions spanning 88,191 bp and 15,750 bp, respectively, and each IR region measuring 29,962 bp (Figure 2). The overall GC content was 36.04%, with regional variations of 33.84% in the LSC, 29.57% in the SSC, and 40.98% in the IR regions. A total of 134 genes were annotated, comprising 86 protein‐coding genes, 40 tRNA genes, and eight rRNA genes. Based on their putative functions, these genes were classified into four categories: 77 genes related to self‐replication, 47 genes involved in photosynthesis, six genes with other functions, and four conserved open reading frames of unknown functions (Table 2). Comparative plastome analysis with four closely related species revealed a high degree of conservation in genome size, GC content, and gene number, with minor variations in the length of individual genomic regions (Table 1).

**FIGURE 2:**
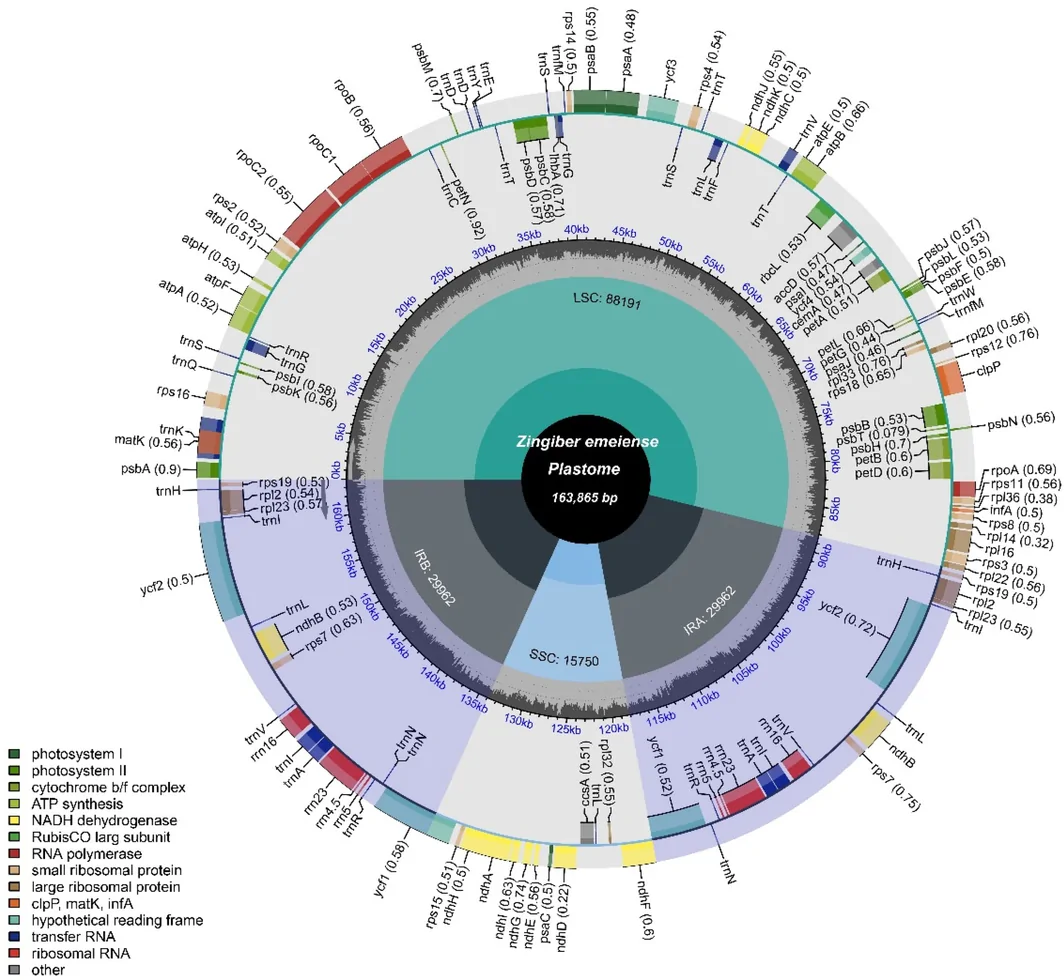
Circular map of the plastome of *Zingiber emeiense*. Annotated genes are displayed around the map and are color‐coded by functional category as indicated in the legend. Genes on the outer circle are transcribed in the counterclockwise direction, while those on the inner circle are transcribed clockwise.

**TABLE 2:** Genetic classification of the plastome of *Zingiber emeiense*.

Category of genes	Group of genes	Name of genes
Self replication	tRNA genes	*trn*A‐UGC(2), *trn*C‐GCA, *trn*D‐GUC(2), *trn*E‐UUC, *trn*F‐GAA, *trn*G‐GCC*(2), *trn*H‐GUG(2), *trn*I‐CAU(2), *trn*I‐GAU*(2), *trn*K‐UUU*, *trn*L(3), *trn*L‐UAA*, *trn*N‐GUU(3), *trn*Q‐UUG, *trn*R‐ACG(2), *trn*R‐UCU, *trn*S(3), *trn*T(3), *trn*V‐GAC(2), *trn*V‐UAC, *trn*W‐CCA, *trn*Y‐GUA, *trn*fM‐CAU(2)
	Small subunit of ribosome	*rps11, *rps*12**, *rps*14, *rps*15, *rps*16*, *rps*18, *rps*19(2), *rps*2, *rps*3, *rps*4, *rps*7(2), *rps*8*
	RNA polymerase	*rpo*A, *rpo*B, *rpo*C1, *rpo*C2*
	Large subunit of ribosome	*rpl*14, *rpl*16, *rpl*2(2), *rpl*20, *rpl*22, *rpl*23(2), *rpl*32, *rpl*33, *rpl*36
	rRNA genes	*rrn*16(2), *rrn*23(2), *rrn*4.5(2), *rrn*5(2)
Photosynthesis	Subunits of photosystem II	*lhb*A, *psb*A, *psb*B, *psb*C, *psb*D, *psb*E, *psb*F, *psb*H, *psb*I, *psb*J, *psb*K, *psb*L, *psb*M, *psb*N, *psb*T
	Subunits of ATP synthase	*atp*A, *atp*B, *atp*E, *atp*F, *atp*H, *atp*I*
	Subunits of cytochrome b/f complex	*pet*A, *pet*B, *pet*D, *pet*G, *pet*L, *pet*N
	Subunits of photosystem I	*psaA, *psa*B, *psa*C, *psa*I, *psa*J, *ycf*3**, *ycf*4*
	Subunits of NADH‐dehydrogenase	*ndh*A, *ndh*B(2), *ndh*C, *ndh*D, *ndh*E, *ndh*F, *ndh*G, *ndh*H, *ndh*I, *ndh*J, *ndh*K
	Large subunit of rubisco	*rbc*L
Other genes	Maturase	*mat*K
	Subunit of acetyl‐coA‐carboxylase	*acc*D
	Carbon metabolism	*cem*A
	Protease	*clp*P**
	Translational initiation factor	*inf*A
	c‐type cytochrom synthesis gene	*ccs*A
Unknown function	Conserved Open Reading Frames	*ycf*1(2), *ycf*2(2)

Abbreviations: Gene*, gene with one intron; Gene**, gene with two introns; Gene (2), number of copies of multi‐copy genes.

Intron analysis revealed that 23 genes contained introns, of which three genes (*rps*12, *clp*P, *ycf*3) harbored two introns and the remaining 20 genes possessed a single intron. On the basis of their splicing patterns, these intron‐containing genes were further categorized into cis‐ and trans‐spliced types (Figure S1). All single‐intron genes, together with the two multi‐intron genes (*ycf*3 and *clp*P), were characterized as cis‐spliced genes (Figure S1a). Notably, *rps*12 was the sole trans‐spliced gene in the plastome of *Z. emeiense*, with its three exons dispersed across distinct genomic regions (Figure S1b).

### 3.2 Codon Usage Bias

Codon usage in the plastome of *Z. emeiense* exhibited a pronounced A/U bias (Table S2). Thirty‐one codons had RSCU values greater than 1, of which 29 ended in A or U, whereas the 31 codons with RSCU < 1 mostly ended in G or C. AGA (Arg, RSCU = 1.9507) and UUA (Leu, RSCU = 1.9098) were the most strongly preferred codons, and AGC (Ser, RSCU = 0.3047) was the least used. Among the three stop codons, UAA was preferred (RSCU = 1.7586) over UAG (0.7759) and UGA (0.4655). Nearly identical codon usage profiles were revealed across the five species (Table S2, Figure S2), with AGA (RSCU = 1.9507–2.0136) and UUA (1.8778–1.9098) consistently the most preferred, AGC (0.2958–0.3055) the least used, and UAA the preferred stop codon in all species. The only difference detected was the marginal overuse of UCC (Ser) in *Z. emeiense*, *Z. atroporphyreum*, and *Z. ellipticum* (RSCU = 1.0036–1.0129) relative to its slight underuse in *Z. mioga* and *Z. officinale* (0.9964–0.9974). Codon usage bias is highly conserved among *Zingiber* plastomes and cannot distinguish *Z. emeiense* from its congeners (Figure S2), in line with the structural conservation of these plastomes.

### 3.3 Variation in IR Boundaries, Repeat Sequences, Sequence Divergence and Nucleotide Diversity

Comparative analysis of the IR boundary regions across five *Zingiber* plastomes revealed an overall conserved evolutionary pattern alongside species‐specific structural variations (Figure 3). Interspecific differences were detected at the LSC/IRb boundary, where the junction was consistently situated within the *rpl*22 in all species, but the length of its extension into the IRb region varied substantially, from 26 bp in *Z. emeiense* to 106 bp in *Z. ellipticum*. In contrast, the IRb/SSC boundary was highly conserved across all taxa, being consistently located near the *ndh*F gene with the IRb region extending 8–28 bp into the SSC region. The SSC/IRa boundary spanned the *ycf*1 gene, which comprised 1554–1595 bp in the SSC region and 5447–5561 bp in the IRa region. The IRa/LSC boundary was structurally consistent across species, located between the *rps*19 and *trn*H genes, with the *trn*H gene positioned 112–125 bp from the junction.

**FIGURE 3:**
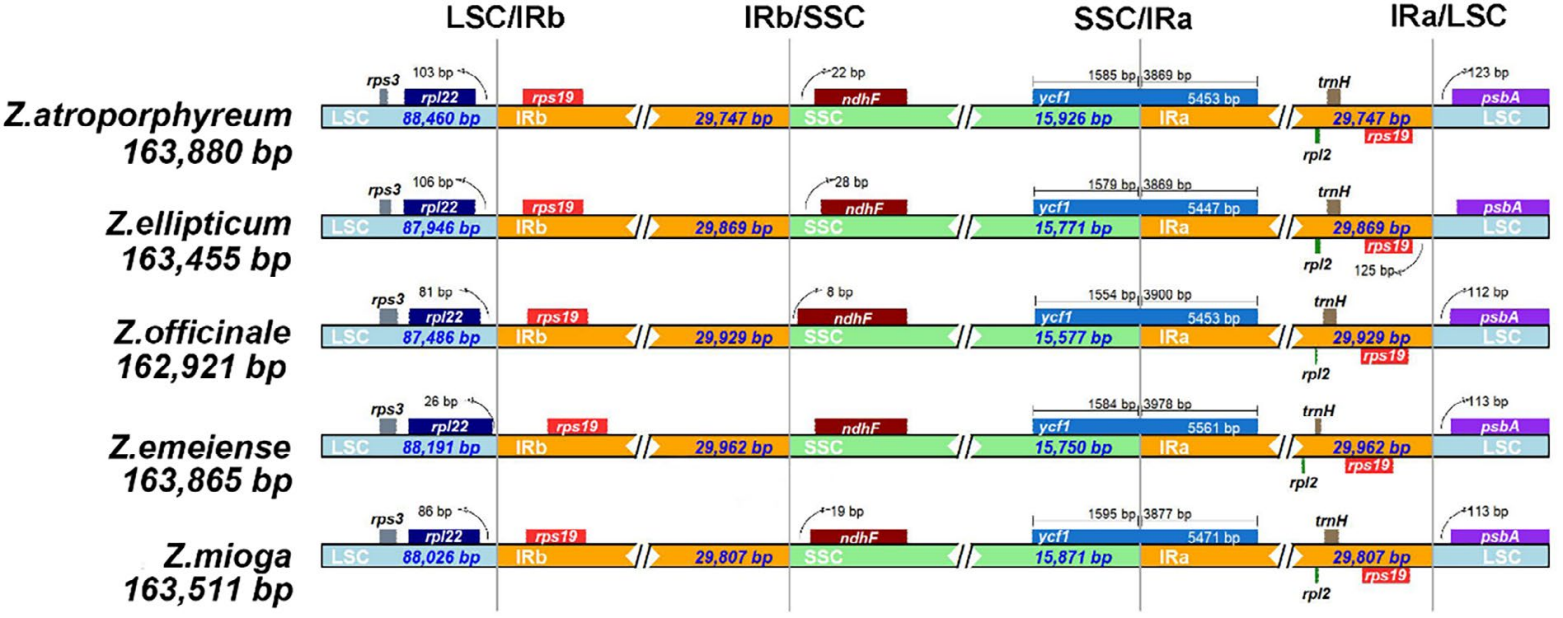
Comparison of LSC, SSC, and IR region boundaries in five *Zingiber* plastomes.

A total of 309 dispersed repeats were identified in the five examined *Zingiber* species (Table S3). Among them, *Z. ellipticum* harbored the most dispersed repeats (72), while *Z. officinale* had the fewest (54). Palindromic repeats were the most abundant type, with 150 copies detected, accounting for 48.5% of all dispersed repeats. In contrast, reverse and complement repeats represented only 9.4% and 0.6% of the total, respectively. Complement repeats were particularly rare, occurring only once in each of *Z. officinale* and *Z. ellipticum*. Notably, among the five species, only *Z. officinale* and *Z. ellipticum* contained all four types of dispersed repeats (Figure 4a,b).

**FIGURE 4:**
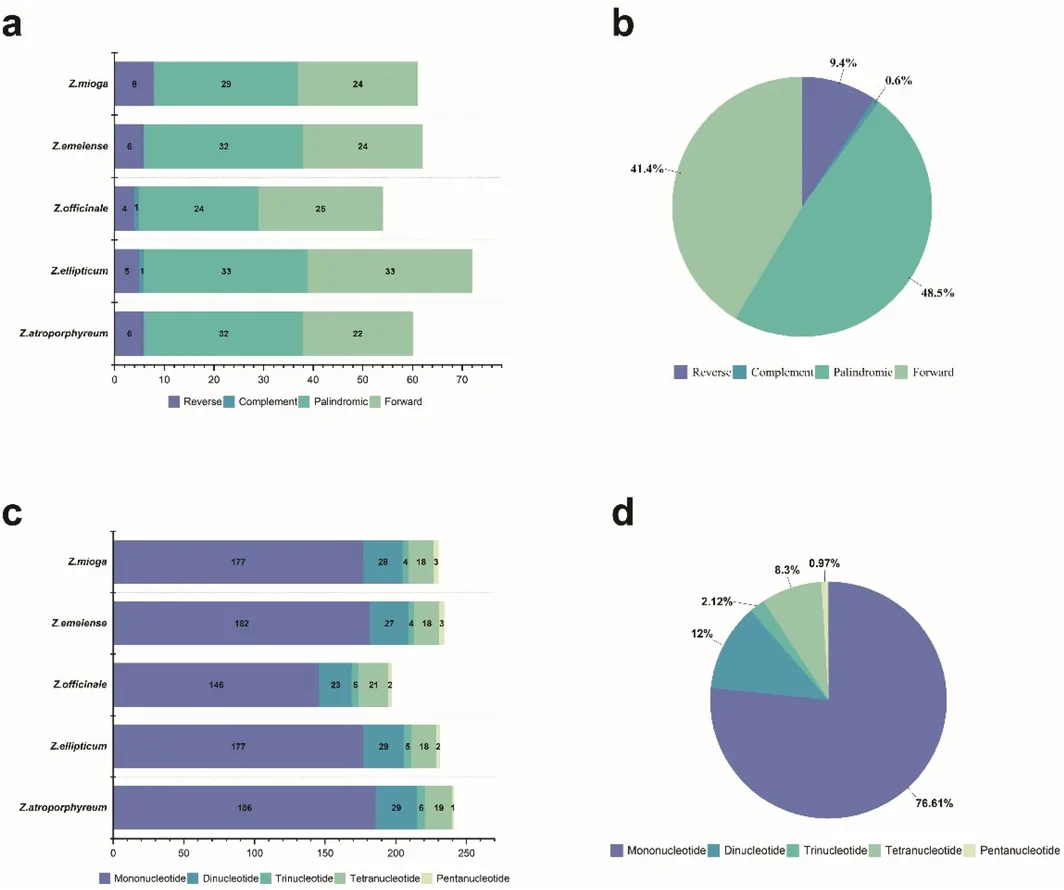
Characteristics of repetitive sequences in the plastomes of five *Zingiber* species. (a) Number of dispersed repeats; (b) proportional distribution of different dispersed repeat types; (c) number of SSRs; (d) proportional distribution of different SSR types.

A total of 1133 SSRs were identified across the five *Zingiber* species (Table S4). All five types of SSRs were present in each of the plastomes. Mononucleotide repeats were the most abundant, accounting for 868 (76.61%) of all SSRs, followed by dinucleotide (136, 12.01%), tetranucleotide (94, 8.30%), and trinucleotide (24, 2.12%) repeats. Pentanucleotide repeats were the least common, accounting for only 0.97% of the total SSRs (Figure 4c,d).

The mVISTA analysis of five *Zingiber* plastomes revealed that gene content and order were highly conserved, with only minor variations detected in boundary regions (Figure S3). Most genomic regions shared more than 90% similarity. However, significant differences were observed among functional regions, with non‐coding sequences (NCSs) and untranslated regions (UTRs) displaying greater variability than exons. The LSC region exhibited the highest level of variation, followed by the SSC region, whereas the IR regions were the most conserved. Collectively, the five *Zingiber* plastomes were highly conserved in structure but exhibited distinct levels of variability among different regions.

The *π* values across all regions ranged from 0 to 0.0399 (Figure 5). Comparison of the three major structural regions (LSC, IR, and SSC) across the five *Zingiber* species showed that the LSC region had the highest nucleotide diversity (*π* = 0.0069), followed by the SSC region (*π* = 0.00637), while the IR region had the lowest (*π* = 0.0037). The *rps*19 gene exhibited the highest value (0.0399), making it the most genetically variable region among five *Zingiber* plastomes. Using π > 0.02 as the threshold—approximately three times the highest regional mean diversity observed in our dataset—we identified seven mutation hotspot regions, including *psb*M, *trn*T‐UGU, *ycf*1, *pet*A‐*psb*J, *ndh*F, *psa*C‐*ndh*E, and *rps*19.

**FIGURE 5:**
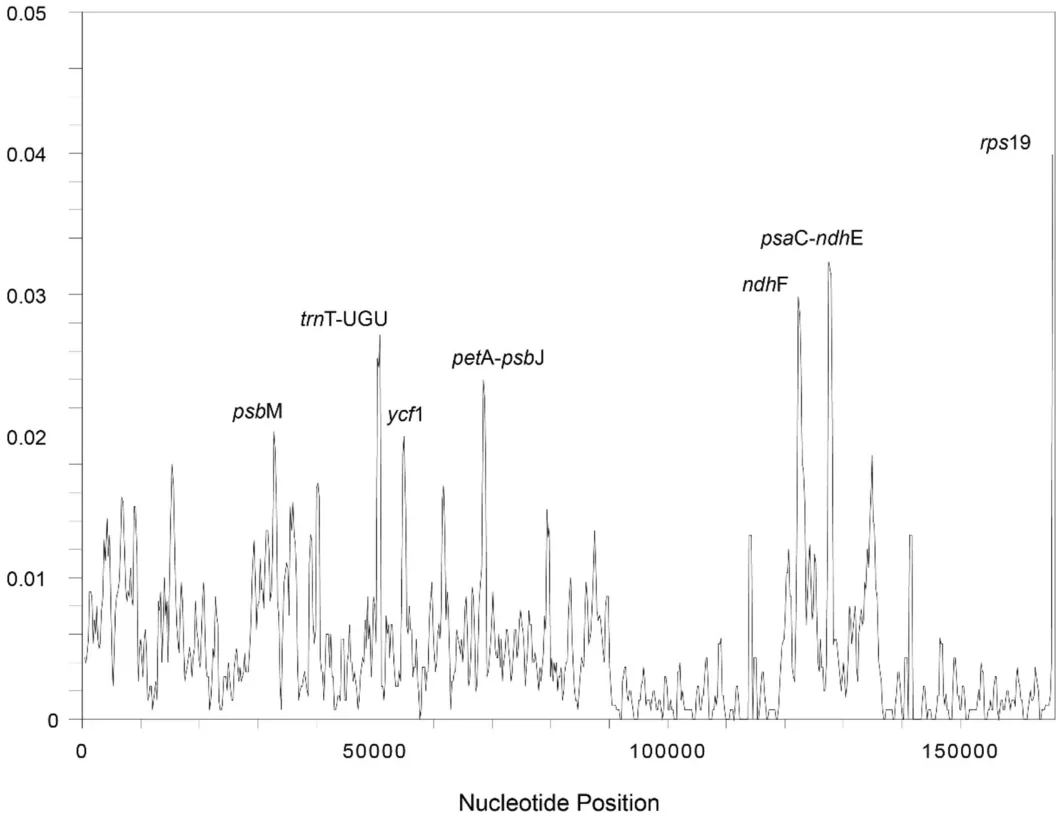
Nucleotide diversity among five *Zingiber* species plastomes.

### 3.4 Selective Evolutionary Pressure

The plastome was found to evolve predominantly under purifying selection, with the mean *ω* of most genes substantially below unity (median = 0.21), consistent with the strong functional constraints characteristic of plastid genomes (Table S5). Nevertheless, ten genes exhibited statistically supported signatures of positive selection. Six of these (*ycf*1, *rpl*20, *ycf*2, *acc*D, *ndh*A, and *pet*D) were under positive selection supported at the gene‐wide (BUSTED *q* < 0.05; *q* denotes the Benjamini–Hochberg‐adjusted *p* value), branch (aBSREL corrected *p* < 0.05), and site levels (MEME *p* < 0.05). *Rpl*2 was were under positive selection at the gene‐wide (BUSTED *q* = 0.031) and branch levels (aBSREL corrected *p* < 0.05), *rbc*L was supported at the branch (aBSREL corrected *p* = 7.6 × 10^−4^) and site levels (MEME *p* = 9 × 10^−4^ and 1.7 × 10^−3^ at codons 19 and 456), and *ndh*F (MEME *p* = 0.048) and *rpo*C2 were supported at the site level (MEME *p* = 5.9 × 10^−3^). Notably, *ycf*1, *pet*D, and *rpl*2 exhibited significant episodic positive selection on the terminal branch of *Z. emeiense* (aBSREL corrected *p* < 10^−16^, *p* = 1.1 × 10^−4^, and *p* = 4.5 × 10^−5^, respectively).

### 3.5 Phylogenetic Relationships

Phylogenetic trees reconstructed using the maximum likelihood (ML) method yielded well‐supported topologies among the 54 species (represented by 70 plastome accessions) examined. *Zingiber* species formed a well‐supported monophyletic group (bootstrap, BS = 100) sister to *Kaempferia* (Figure 6). Within *Zingiber*, sect. *Pleuranthesis* was resolved as the earliest‐diverging lineage, consisting solely of *Z. ellipticum*. The remaining species were further divided into two major clades. One clade corresponded to sect. *Cryptanthium* (BS = 100). Our target species *Z. emeiense* was placed within this section. Two individuals of *Z. emeiense* formed a well‐supported clade that was sister to the clade of *Z. smilesianum* and *Zingiber teres* S. Q. Tong & Y. M. Xia (BS = 100). The other clade comprised nine species from sect. *Zingiber* and sect. *Dymczewiczia*, while *Z. atroporphyreum* was recovered as the sole representative of sect. *Dymczewiczia*.

**FIGURE 6:**
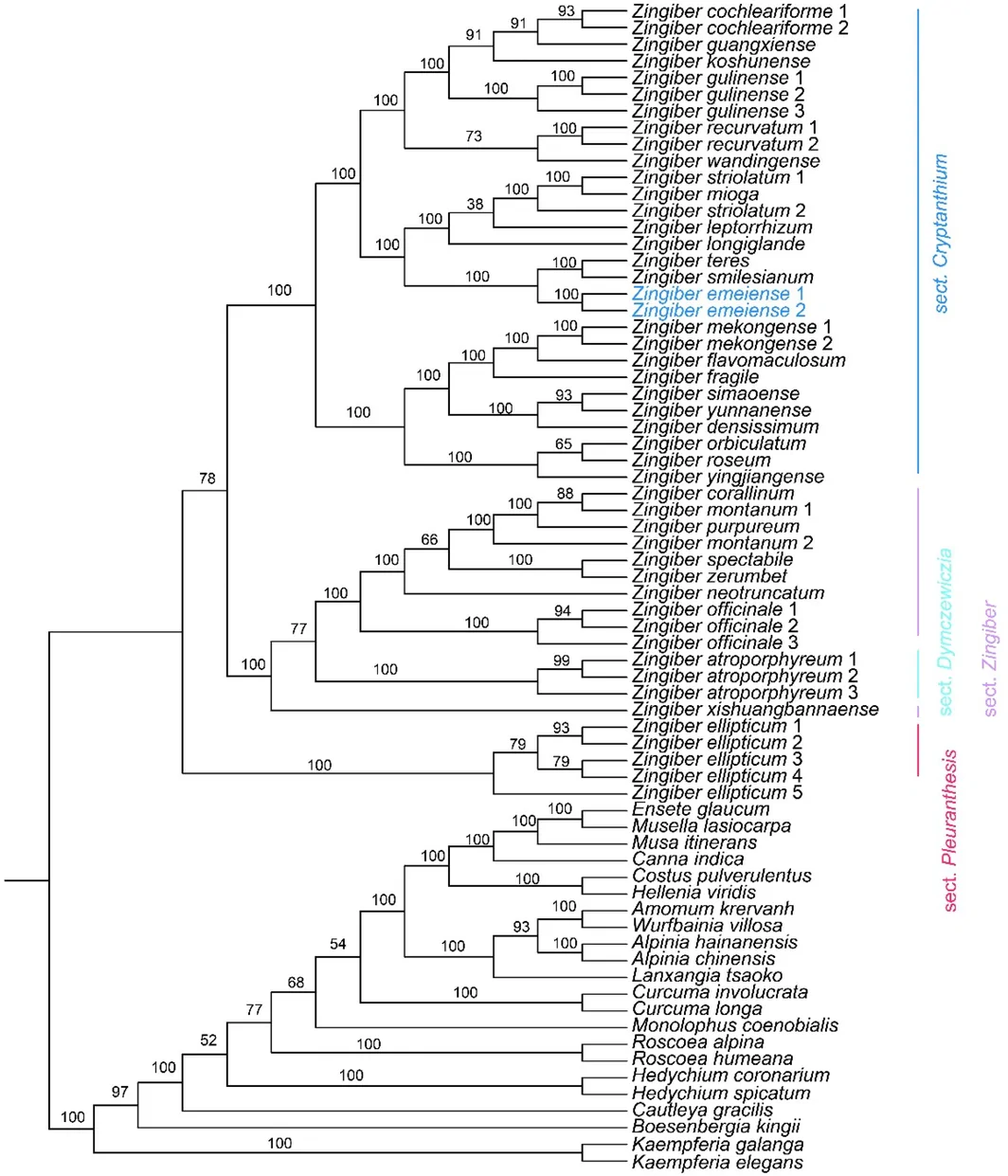
ML phylogenetic tree of *Zingiber* based on 70 complete plastomes. Values at the nodes indicate maximum likelihood bootstrap support (BS); branch lengths are shown, and different colors denote distinct clustering clades.

## 4 Discussion

### 4.1 Structural Characteristics of the Plastome of *Zingiber emeiense*

Plastomes are highly valuable in plant phylogenetic research, primarily attributable to their relatively conserved structure and uniparental (typically maternal in angiosperms) inheritance pattern (Tsunewaki and Ogihara 1983; Xi et al. 2022; Al‐Andal 2025; Yadav et al. 2025). In this study, we successfully assembled and annotated the first two plastomes of *Z. emeiense*. The two plastomes of *Z. emeiense* were identical in sequence and structure, each 163,865 bp in length and consisting of a LSC region, a SSC region, and a pair of IR regions. A total of 134 functional genes were annotated, with an overall GC content of 36.04%. Consistent with previous researches, the plastome of *Z. emeiense* exhibited a highly conserved structure with no evidence of gene rearrangement or loss, further confirming the structural conservation of plastomes within the genus *Zingiber* (Jiang et al. 2023; Xia et al. 2024).

Codon usage bias analysis revealed a pronounced preference pattern. For example, arginine exhibited a marked preference for AGA, while leucine showed a preference for UUA (Table S2). Additionally, almost all preferred codons (29 of 31) in the *Z. emeiense* plastome terminated with A or U, exhibiting a pronounced A/U bias—a pattern consistent with the codon usage in the plastome of most terrestrial plants (Liu et al. 2021). Such codon usage patterns may enhance the translation efficiency of chloroplast genes, thereby contributing to plant adaptability under selective pressure (Parvathy et al. 2022; Robbins and Kelly 2023; Liu et al. 2025).

### 4.2 Comparative Plastome Analysis of *Zingiber* Species

Boundary expansion and contraction in plastomes were common evolutionary phenomena in angiosperms and served as important sources of genomic variation (Wang et al. 2008). Although the five *Zingiber* species investigated in this study displayed highly conserved genome structures, distinct interspecific differences were detected at the LSC/IRb boundary, while the IRb/SSC boundary remained highly conserved (Figure 3). These boundary variations suggest that the IR expansion and contraction in *Zingiber* plastomes are subject to strong evolutionary constraints, maintaining an overall highly conserved structural organization (Jiang et al. 2023; Xia et al. 2024; Lu et al. 2025). Beyond these structural patterns, the observed boundary differences have several biological implications. First, junction shifts directly alter gene dosage, because IR expansion incorporates adjacent single‐copy sequences into the repeat (Wang et al. 2008). The 3′ portion of *rpl*22, for instance, extends 26 bp into IRb in *Z. emeiense* but 106 bp in *Z. ellipticum*, such that a larger fragment of this gene is present in two copies in the latter. Second, the two IR copies undergo copy correction and therefore accumulate substitutions more slowly than single‐copy regions; boundary movements modify the evolutionary‐rate regime experienced by junction‐spanning genes (Wicke et al. 2011). This is particularly relevant for *ycf*1, which straddles the SSC/IRa junction with most of its length residing in the IRa copy, so that shifts in junction position alter the relative proportions of this gene evolving under IR constraint and under single‐copy conditions. Third, the interspecific length differences at the LSC/IRb junction represent lineage‐specific expansion events that constitute a principal source of plastome size variation in the genus. In addition, the number and type of dispersed repetitive sequences and SSRs also affected genome sequence length, which are important molecular markers for studies of genetic diversity, population structure, and evolutionary relationships (Ebert and Peakall 2009; George et al. 2015). A total of 1133 SSRs and 309 dispersed repeats were identified across the five *Zingiber* accessions, with clear variation in the types and numbers of repeat sequences among different species (Tables S3 and S4). These SSRs and repetitive sequences represent valuable genomic resources for *Zingiber* and will facilitate assessments of genetic diversity, germplasm identification, and molecular marker‐assisted breeding in this genus (Li et al. 2025).

Consistent with the prevailing view of plastome evolution (Wicke et al. 2011; Daniell et al. 2016), purifying selection constituted the dominant evolutionary force shaping *Zingiber* plastomes. Nonetheless, likelihood‐based codon models identified ten plastid genes with signatures of positive selection among the 32 *Zingiber* species, six of which (*ycf*1, *rpl*20, *ycf*2, *acc*D, *ndh*A, and *pet*D) were consistently supported across gene‐wide, branch‐level, and site‐level tests. Recurrent positive selection on plastid genes, including *ycf*1, *ycf*2, *rbc*L, and *acc*D, has also been documented in other angiosperm lineages, implying that a restricted repertoire of plastid genes repeatedly undergoes adaptive evolution during lineage diversification (Frazão et al. 2023; Campos et al. 2026; Li et al. 2020).

Of particular note, *ycf*1, *pet*D, and *rpl*2 exhibited significant positive selection specifically along the terminal branch of *Z. emeiense*, indicative of lineage‐specific adaptive changes in this species. *Ycf*1 encodes Tic214, an essential core component of the 1‐MDa protein translocation complex at the inner envelope membrane of chloroplasts, which mediates the import of thousands of nucleus‐encoded photosynthetic proteins (de Vries et al. 2015; Kikuchi et al. 2018). *Pet*D encodes subunit IV of the cytochrome b6f complex, the rate‐limiting electron carrier that couples electron flow to proton translocation and governs photosynthetic efficiency under fluctuating light conditions (Baniulis et al. 2008). *Rpl*2 encodes ribosomal protein L2 of the plastid 50S subunit, which is indispensable for plastid translation and, consequently, for the biogenesis of the entire photosynthetic apparatus (Tiller and Bock 2014). Given that *Z. emeiense* grows in shaded, humid montane understory of Mount Emei, positive selection on genes governing photosynthetic electron transport (*pet*D), plastid translation (*rpl*2), and protein import (*ycf*1) may reflect fine‐tuning of photosynthetic performance and proteostasis during adaptation to this specialized habitat. However, such functional inferences remain speculative in the absence of physiological validation. These lineage‐specific signatures provide concrete molecular targets for future investigations into the adaptive evolution of *Z. emeiense*.

### 4.3 Phylogenetic Relationships and Taxonomic Implications

We performed phylogenetic analyses using 70 plastomes (representing 54 species) to reconstruct a robust phylogenetic framework for *Zingiber* and to determine the phylogenetic position of *Z. emeiense* for the first time. The resulting phylogeny recovered three well‐supported monophyletic clades within the genus, corresponding to sect. *Pleuranthesis*, sect. *Zingiber* + sect. *Dymczewiczia*, and sect. *Cryptanthium*, a topology congruent with previous taxonomic treatments based on inflorescence habit and pollen morphology (Jiang et al. 2023; Xia et al. 2024; Wang et al. 2026). *Zingiber emeiense* was placed within sect. *Cryptanthium* as a sister clade to *Z. teres* and *Z. smilesianum* (Figure 6).

Previous studies have confirmed that *Z. smilesianum* and *Z. teres* are conspecific synonyms (Bai 2016). In this study, we compared *Z. emeiense*, *Z. teres*, and *Z. striolatum* with respect to three morphological characters, namely bract shape, corolla lobe color, and labellum color, all of which differed distinctly among the three species (Xiao 2016). Specifically, *Z. emeiense* has oblong‐lanceolate to lanceolate bracts, whereas *Z. teres* possesses narrowly ovate to linear bracts that are longitudinally involute and sub‐tubular, and *Z. striolatum* bears broadly ovate to elliptic bracts (Zhu 1984; Bai 2016). Corolla lobes are vermilion in *Z. emeiense*, golden‐yellow in *Z. teres*, and white to slightly yellowish in *Z. striolatum* (Souvannakhoummane and Leong‐Škorničková 2018; Wang et al. 2023). The labellum is bluish‐purple to reddish‐purple in *Z. emeiense*, orange‐red to dark reddish‐purple with yellow stripes in *Z. teres*, and light purple in *Z. striolatum* (Wu and Larsen 2000; Bai 2016). Collectively, these morphological traits provide reliable diagnostic characters for distinguishing the three species.

The deep phylogenetic split between *Z. emeiense* and *Z. striolatum* carries broader evolutionary and taxonomic implications. In Flora of China, *Z. emeiense* was recognized as a synonym of *Z. striolatum* (Wu and Larsen 2000). Due to the morphologic differences or the results of phylogenetic analysis (see above), we do not support this classification. The morphological similarity that originally motivated the synonymy, especially the basal and prostrate‐peduncled inflorescence characteristic of sect. *Cryptanthium*, reflects the common structure shared by the section rather than intra‐specific consistency. Therefore, inflorescence characters alone are unreliable for species delimitation in *Zingiber* (Kress et al. 2002; Theerakulpisut et al. 2012). The distant placement of *Z. striolatum* relative to *Z. emeiense* further indicates that broad, morphology‐based species concepts in *Zingiber* may mask cryptic diversity. These results demonstrate that complete plastomes, by providing genome‐scale characters independent of morphology, offer a robust basis for re‐evaluating long‐standing synonymies and species boundaries in taxonomically difficult genera (Song et al. 2020; Wang et al. 2021; Jiang et al. 2023; Xia et al. 2024). For *Z. emeiense*, its resolution as a distinct, fully supported lineage endemic to Mount Emei establishes it as an independent evolutionary lineage and, accordingly, a separate unit for conservation.

The genomic results are also consistent with the ecological and morphological distinctness of *Z. emeiense*. Unlike most congeners, which occur in lowland rainforests, *Z. emeiense* is confined to moist, shaded understory habitats at mid‐elevations (ca. 900 m a.s.l.) on a single massif (Fang 1982; Bai et al. 2018). And its elongate creeping rhizome—shared with its sister species—facilitates clonal persistence on the forest floor (Bai 2016). Long‐term ecological isolation on Mount Emei, combined with the predominantly maternal inheritance and slow nucleotide substitution rates of the plastome, provides a plausible context for the origin and maintenance of this distinct lineage (Tsunewaki and Ogihara 1983; Wicke et al. 2011). The two plastomes of *Z. emeiense* independently assembled and annotated in this study are identical in sequence and structure, which is consistent with the characteristic of the low nucleotide substitution rate of chloroplasts.

However, in the absence of environmental data, any adaptive interpretation linking plastome variation to these ecological settings remains speculative. However, population‐level variation of *Z. emeiense* cannot be assessed from two accessions. To test these ecological evolution hypotheses, a larger population sample size is required, and phylogeographic research could be conducted using the variable regions or SSR sequences identified in this study.

## 5 Conclusions

This study reported the first two complete plastomes of *Z. emeiense* and provided comprehensive comparative genomic and phylogenomic analyses alongside representative *Zingiber* taxa. Consistent with its congeners, the plastome of *Z. emeiense* exhibited a typical quadripartite structure with high structural conservation. Inverted repeat boundary dynamics and repetitive sequences were key drivers of genome size variation within *Zingiber*. Seven hypervariable regions were identified as potential molecular markers for species identification and population genetic studies. The plastome of *Z. emeiense* exhibited a strong A/U bias in codon usage. Chloroplast gene evolution across the genus *Zingiber* was mainly shaped by purifying selection, although several genes showed lineage‐specific positive selection signals. Phylogenomic analysis placed *Z. emeiense* within sect. *Cryptanthium*, as a sister to the clade of *Z. teres* and *Z. smilesianum*.

Collectively, we resolved the long‐standing taxonomic ambiguity between *Z. emeiense* and *Z. striolatum*, and enriched the genomic resources of *Zingiber*. *Zingiber emeiense* formed a distinct, fully supported lineage clearly separated from *Z. striolatum*, supporting its status as a valid species and independent conservation target. The seven hypervariable regions (e.g., *rps*19, *ycf*1, and *ndh*F) constitute candidate molecular markers for unambiguous discrimination of *Z. emeiense* from its congeners, providing a technical basis for field identification and germplasm authentication. Furthermore, the plastome‐derived SSRs and hypervariable loci furnish transferable markers for population‐level assessment of genetic diversity, which will inform both in situ management and ex situ germplasm preservation.

## Author Contributions

**Hualan Chen:** conceptualization (lead), formal analysis (lead), methodology (lead), writing – original draft (lead). **Zhaofei Wang:** data curation (equal), investigation (equal), validation (equal). **Jinyong Ni:** software (equal), visualization (equal). **Qinxi Feng:** data curation (equal), investigation (equal). **Yue Liu:** data curation (equal), validation (equal). **Lihui Jiang:** writing – review and editing (equal). **Chao Song:** writing – review and editing (equal). **Yanlin Zhu:** investigation (equal), resources (equal). **Menghan Zhang:** data curation (equal), visualization (equal). **Qin Xie:** investigation (equal), validation (equal). **Maoqin Xia:** conceptualization (equal), funding acquisition (equal), resources (equal), supervision (equal). **Honglei Li:** conceptualization (equal), funding acquisition (equal), supervision (equal), writing – review and editing (equal).

## Funding

This work was supported by the National Natural Science Foundation of China (32600315, 32270237), the Natural Science Foundation of Chongqing (CSTB2025NSCQ‐GPX0340), the Science and Technology Research Program of Chongqing Municipal Education Commission (KJQN202401310), the Foundation for High‐level Talents of Chongqing University of Arts and Sciences (R2022YS09), and the Postgraduate Research Innovation Projects of Chongqing University of Arts and Sciences (CUAS‐GSK2025019, CUAS‐GSK2025021).

## Ethics Statement

The collection of plant samples was conducted in compliance with relevant environmental regulations, ensuring no harm to the local ecosystem.

## Conflicts of Interest

The authors declare no conflicts of interest.

## Supporting information


**Figure S1:** Gene structure map of cis‐ and trans‐spliced genes in the *Z. emeiense* plastome.
**Figure S2:** Codon usage bias in protein ‐coding genes of five *Zingiber* species.
**Figure S3:** Pairwise plastome alignment of five *Zingiber* species.
**Table S1:** Plastome sequences used in this study.
**Table S2:** Codon usage bias analysis of the five *Zingiber* species.
**Table S3:** Summary of dispersed repeat types identified in the plastomes of five *Zingiber* species.
**Table S4:** Summary of SSR types identified in the plastomes of five *Zingiber* species.
**Table S5:** HyPhy selection analysis results for all 74 shared plastid protein‐coding genes among 32 *Zingiber* species.

## Data Availability

The genome sequence data supporting the findings of this study are available in GenBank (https://www.ncbi.nlm.nih.gov/) under the accession numbers PZ174020 and PZ174021.
