# Supplementary material for: Characterization of the First Complete Plastome of Zingiber emeiense: Insights Into Its Phylogenetic Position
Source: Ecol Evol. 2026 Oct 25;16(10):e74465. doi: 10.1002/ece3.74465 (PMC13710891; doi:10.1002/ece3.74465)
Supplement: Supplementary file 1 [file ECE3-16-e74465-s001.docx]

**Characterization of the first complete chloroplast genome of *Zingiber emeiense*: insights into its phylogenetic position**

Hualan Chen^1^, Zhaofei Wang^1^, Jinyong Ni^1^, Qinxi Feng^1^, Yue Liu^1^, Lihui Jiang^1,2^, Chao Song^1,2^, Yanlin Zhu^1^, Menghan Zhang^1^, Qin Xie^1^, Maoqin Xia^1,2*^ and Honglei Li^1,2*^

**Figure S1.** Gene structure map of cis- and trans-spliced genes in the *Z. emeiense* plastome. (a) Cis-spliced genes. (b) Trans-spliced genes.

**Figure S2.** Codon usage bias in protein -coding genes of five *Zingiber* species*.*

**Figure S3.** Pairwise plastome alignment of five *Zingiber* species.

**Table S1.** Plastome sequences used in this study.

| Taxon | Accession nunber | Genome size(bp) |
| --- | --- | --- |
| *Zingiber cochleariforme* 1 | OP869986 | 163,665 |
| *Zingiber cochleariforme* 2 | N_001486764 | 162,855 |
| *Zingiber guangxiense* | OR337870 | 163,050 |
| *Zingiber koshunense* | OP869977 | 163,394 |
| *Zingiber gulinense* 1 | N_001486770 | 162,790 |
| *Zingiber gulinense* 2 | N_001486773 | 162,790 |
| *Zingiber gulinense* 3 | N_001486771 | 162,791 |
| *Zingiber recurvatum* 1 | OR337876 | 163,129 |
| *Zingiber recurvatum* 2 | N_001486777 | 163,162 |
| *Zingiber wandingense* | OR337879 | 163,398 |
| *Zingiber striolatum* 1 | N_001486778 | 163,611 |
| *Zingiber mioga* | OR337872 | 163,511 |
| *Zingiber striolatum* 2 | ON646165 | 163,711 |
| *Zingiber leptorrhizum* | OP869984 | 162,956 |
| *Zingiber longiglande* | OR337871 | 163,125 |
| *Zingiber teres* | NC_062457 | 163,428 |
| *Zingiber smilesianum* | OP869981 | 163,640 |
| *Zingiber emeiense* 1 | PZ174020 | 163,865 |
| *Zingiber emeiense* 2 | PZ174021 | 163,865 |
| *Zingiber mekongense* 1 | N_001486774 | 163,261 |
| *Zingiber mekongense* 2 | N_001486775 | 163,309 |
| *Zingiber flavomaculosum* | OP869987 | 163,481 |
| *Zingiber fragile* | OR337869 | 163,381 |
| *Zingiber simaoense* | OR337878 | 163,551 |
| *Zingiber yunnanense* | OR337880 | 163,772 |
| *Zingiber densissimum* | OP869975 | 163,607 |
| *Zingiber orbiculatum* | OP869979 | 163,527 |
| *Zingiber roseum* | OR337877 | 163,529 |
| *Zingiber yingjiangense* | OP869983 | 163,623 |
| *Zingiber corallinum* | MW801385 | 160,957 |
| *Zingiber montanum* 1 | N_001486776 | 161,495 |
| *Zingiber purpureum* | OP869980 | 163,135 |
| *Zingiber montanum* 2 | OP869985 | 163,476 |
| *Zingiber spectabile* | JX088661 | 155,890 |
| *Zingiber zerumbet* | MK262726 | 163,589 |
| *Zingiber neotruncatum* | OP869978 | 162,481 |
| *Zingiber officinale* 1 | OR337873 | 162,921 |
| *Zingiber officinale* 2 | OR337875 | 162,921 |
| *Zingiber officinale* 3 | OR337874 | 162,921 |
| *Zingiber atroporphyreum* 1 | N_001486761 | 163,880 |
| *Zingiber atroporphyreum* 2 | N_001486763 | 163,880 |
| *Zingiber atroporphyreum* 3 | N_001486762 | 163,879 |
| *Zingiber xishuangbannaense* | OP869982 | 163,487 |
| *Zingiber ellipticum* 1 | N_001486766 | 163,295 |
| *Zingiber ellipticum* 2 | N_001486769 | 163,290 |
| *Zingiber ellipticum* 3 | N_001486767 | 163,413 |
| *Zingiber ellipticum* 4 | N_001486768 | 163,413 |
| *Zingiber ellipticum* 5 | OP869976 | 163,455 |
| *Ensete glaucum* | LC610748 | 168,811 |
| *Musella lasiocarpa* | NC_035637 | 169,178 |
| *Musa itinerans* | NC_035723 | 168,985 |
| *Canna indica* | KF601570 | 162,182 |
| *Costus pulverulentus* | KF601573 | 163,239 |
| *Hellenia viridis* | MK262733 | 168,966 |
| *Amomum krervanh* | MF991963 | 162,766 |
| *Wurfbainia villosa* | MN067433 | 163,981 |
| *Alpinia chinensis* | NC_050165 | 163,590 |
| *Alpinia hainanensis* | MK262728 | 162,387 |
| *Lanxangia tsaoko* | MK926774 | 163,648 |
| *Curcuma involucrata* | MK262725 | 163,300 |
| *Curcuma longa* | MK262732 | 162,176 |
| *Monolophusn coenobialis* | MW326458 | 162,136 |
| *Roscoea alpina* | MT012418 | 163,395 |
| *Roscoea humeana* | NC_046582 | 160,288 |
| *Hedychium coronarium* | MK262736 | 163,949 |
| *Hedychium spicatum* | NC_047248 | 163,531 |
| *Cautleya gracilis* | NC_046845 | 164,001 |
| *Boesenbergia kingii* | MW326451 | 163,198 |
| *Kaempferia elegans* | MK209002 | 163,555 |
| *Kaempferia galanga* | MK209001 | 163,811 |

**Table S2.** Codon usage bias analysis of the five *Zingiber* species.

| **Amino acid** | **Codon** | ***Z. emeiense*** | ***Z. atroporphyreum*** | ***Z. ellipticum*** | ***Z. mioga*** | ***Z. officinale*** |
| --- | --- | --- | --- | --- | --- | --- |
| * | UAA | 1.7586 | 1.6842 | 1.7895 | 1.7368 | 1.7895 |
| * | UAG | 0.7759 | 0.7895 | 0.7368 | 0.7895 | 0.7368 |
| * | UGA | 0.4655 | 0.5263 | 0.4737 | 0.4737 | 0.4737 |
| A | GCA | 1.2775 | 1.2442 | 1.2335 | 1.2506 | 1.2470 |
| A | GCC | 0.6047 | 0.6110 | 0.6183 | 0.6046 | 0.6171 |
| A | GCG | 0.3287 | 0.3309 | 0.3378 | 0.3278 | 0.3325 |
| A | GCU | 1.7891 | 1.8138 | 1.8104 | 1.8170 | 1.8034 |
| C | UGC | 0.4746 | 0.4539 | 0.4523 | 0.4523 | 0.4429 |
| C | UGU | 1.5254 | 1.5461 | 1.5477 | 1.5477 | 1.5571 |
| D | GAC | 0.3556 | 0.3604 | 0.3615 | 0.3585 | 0.3543 |
| D | GAU | 1.6444 | 1.6396 | 1.6385 | 1.6415 | 1.6457 |
| E | GAA | 1.4886 | 1.4820 | 1.4854 | 1.4779 | 1.4910 |
| E | GAG | 0.5114 | 0.5180 | 0.5146 | 0.5221 | 0.5090 |
| F | UUC | 0.7136 | 0.7275 | 0.7242 | 0.7249 | 0.7291 |
| F | UUU | 1.2864 | 1.2725 | 1.2758 | 1.2751 | 1.2709 |
| G | GGA | 1.6624 | 1.6418 | 1.6355 | 1.6576 | 1.6564 |
| G | GGC | 0.3194 | 0.3200 | 0.3202 | 0.3177 | 0.3239 |
| G | GGG | 0.6388 | 0.6449 | 0.6576 | 0.6404 | 0.6329 |
| G | GGU | 1.3793 | 1.3932 | 1.3867 | 1.3842 | 1.3869 |
| H | CAC | 0.4231 | 0.4300 | 0.4262 | 0.4207 | 0.4307 |
| H | CAU | 1.5769 | 1.5700 | 1.5738 | 1.5793 | 1.5693 |
| I | AUA | 0.9904 | 0.9721 | 0.9754 | 0.9772 | 0.9819 |
| I | AUC | 0.5469 | 0.5537 | 0.5510 | 0.5562 | 0.5438 |
| I | AUU | 1.4627 | 1.4742 | 1.4736 | 1.4665 | 1.4743 |
| K | AAA | 1.4848 | 1.4749 | 1.4757 | 1.4780 | 1.4716 |
| K | AAG | 0.5152 | 0.5251 | 0.5243 | 0.5220 | 0.5284 |
| L | CUA | 0.8297 | 0.8155 | 0.8238 | 0.8221 | 0.8148 |
| L | CUC | 0.3936 | 0.3972 | 0.3965 | 0.3934 | 0.3838 |
| L | CUG | 0.3377 | 0.3408 | 0.3399 | 0.3369 | 0.3391 |
| L | CUU | 1.2657 | 1.2926 | 1.2935 | 1.2956 | 1.2975 |
| L | UUA | 1.9098 | 1.8778 | 1.8836 | 1.8846 | 1.8862 |
| L | UUG | 1.2635 | 1.2761 | 1.2628 | 1.2674 | 1.2786 |
| M | AUG | 1.0000 | 1.0000 | 1.0000 | 1.0000 | 1.0000 |
| N | AAC | 0.4658 | 0.4598 | 0.4596 | 0.4593 | 0.4535 |
| N | AAU | 1.5342 | 1.5402 | 1.5404 | 1.5407 | 1.5465 |
| P | CCA | 1.1270 | 1.1411 | 1.1389 | 1.1337 | 1.1509 |
| P | CCC | 0.7488 | 0.7648 | 0.7632 | 0.7598 | 0.7606 |
| P | CCG | 0.4546 | 0.4404 | 0.4436 | 0.4342 | 0.4306 |
| P | CCU | 1.6695 | 1.6537 | 1.6543 | 1.6724 | 1.6579 |
| Q | CAA | 1.5293 | 1.5103 | 1.5097 | 1.5092 | 1.5138 |
| Q | CAG | 0.4707 | 0.4897 | 0.4903 | 0.4908 | 0.4862 |
| R | AGA | 1.9507 | 1.9892 | 1.9986 | 1.9795 | 2.0136 |
| R | AGG | 0.6619 | 0.6793 | 0.6812 | 0.6762 | 0.6617 |
| R | CGA | 1.2966 | 1.2447 | 1.2522 | 1.2541 | 1.2417 |
| R | CGC | 0.3465 | 0.3376 | 0.3304 | 0.3402 | 0.3227 |
| R | CGG | 0.4166 | 0.4108 | 0.4079 | 0.4098 | 0.4084 |
| R | CGU | 1.3277 | 1.3383 | 1.3297 | 1.3402 | 1.3519 |
| S | AGC | 0.3047 | 0.3014 | 0.3055 | 0.3014 | 0.2958 |
| S | AGU | 1.2129 | 1.2336 | 1.2312 | 1.2460 | 1.2517 |
| S | UCA | 1.2334 | 1.2118 | 1.2062 | 1.2211 | 1.2081 |
| S | UCC | 1.0049 | 1.0129 | 1.0036 | 0.9974 | 0.9964 |
| S | UCG | 0.5508 | 0.5438 | 0.5455 | 0.5407 | 0.5387 |
| S | UCU | 1.6934 | 1.6965 | 1.7081 | 1.6934 | 1.7094 |
| T | ACA | 1.2798 | 1.2745 | 1.2690 | 1.2678 | 1.2637 |
| T | ACC | 0.7048 | 0.7092 | 0.7195 | 0.7011 | 0.6988 |
| T | ACG | 0.4730 | 0.4673 | 0.4644 | 0.4685 | 0.4604 |
| T | ACU | 1.5425 | 1.5490 | 1.5470 | 1.5627 | 1.5771 |
| V | GUA | 1.5166 | 1.5330 | 1.5309 | 1.5368 | 1.5389 |
| V | GUC | 0.4346 | 0.4363 | 0.4321 | 0.4356 | 0.4348 |
| V | GUG | 0.5795 | 0.5622 | 0.5648 | 0.5552 | 0.5644 |
| V | GUU | 1.4693 | 1.4685 | 1.4722 | 1.4724 | 1.4618 |
| W | UGG | 1.0000 | 1.0000 | 1.0000 | 1.0000 | 1.0000 |
| Y | UAC | 0.4367 | 0.4205 | 0.4222 | 0.4204 | 0.4254 |
| Y | UAU | 1.5633 | 1.5795 | 1.5778 | 1.5796 | 1.5746 |

**Table S3.** Summary of dispersed repeat types identified in the plastomes of five *Zingiber* species.

| Species | Reverse | Complement | Palindromic | Forward | Total |
| --- | --- | --- | --- | --- | --- |
| *Z.atroporphyreum* | 6 | 0 | 32 | 22 | 60 |
| *Z.ellipticum* | 5 | 1 | 33 | 33 | 72 |
| *Z.officinale* | 4 | 1 | 24 | 25 | 54 |
| *Z.emeiense* | 6 | 0 | 32 | 24 | 62 |
| *Z.mioga* | 8 | 0 | 29 | 24 | 61 |
| Total | 29 | 2 | 150 | 128 | 309 |

**Table S4.** Summary of SSR types identified in the plastomes of five *Zingiber* species.

| Species | Mononucleotide | Dinucleotide | Trinucleotide | Tetranucleotide | Pentanucleotide | Total |
| --- | --- | --- | --- | --- | --- | --- |
| *Z.atroporphyreum* | 186 | 29 | 6 | 19 | 1 | 241 |
| *Z.ellipticum* | 177 | 29 | 5 | 18 | 2 | 231 |
| *Z.officinale* | 146 | 23 | 5 | 21 | 2 | 197 |
| *Z.emeiense* | 182 | 27 | 4 | 18 | 3 | 234 |
| *Z.mioga* | 177 | 28 | 4 | 18 | 3 | 230 |
| Total | 868 | 136 | 24 | 94 | 11 | 1133 |

**Table S5** HyPhy selection analysis results for all 74 shared plastid protein-coding genes among 32 *Zingiber* species. Mean *ω*, site-proportion-weighted mean dN/dS across the tree (MG94 baseline model); *q* (BH), Benjamini-Hochberg adjusted *P* value; *ω*+ (prop.), the *ω* value and site proportion of the positive-selection rate class (*ω* > 1) inferred by BUSTED; aBSREL sig. branches, number of branches with corrected *P* < 0.05; MEME sig. sites / FEL pos. sites, number of codon sites with *P* < 0.05 (FEL counts only sites with dN > dS); *Z. emeiense* branch, corrected *P* value of the aBSREL test for positive selection on the terminal branch of *Z. emeiense*; shown only for genes with *P* < 0.05 (*pet*D, *rpl*2 and *ycf*1).

| **Gene** | **Codons** | **Mean *ω*** | **BUSTED *P*** | ***q* (BH)** | ***ω*+ (prop.)** | | **aBSREL sig. branches** | **MEME sig. sites** | **FEL pos. sites** | ***Z. emeiense* branch** | **Evidence** |
| --- | --- | --- | --- | --- | --- | --- | --- | --- | --- | --- | --- |
| *acc*D | 657 | 0.980 | 3.7e-07 | 6.8e-06 | 39.08 (2.7%) | | 1 | 2 | 1 | — | **gene-wide/branch/site** |
| *atp*A | 507 | 0.094 | 0.500 | 0.500 | 5.12 (0.0%) | | 0 | 0 | 0 | — | No |
| *atp*B | 495 | 0.188 | 0.500 | 0.500 | 1.00 (0.0%) | | 0 | 0 | 0 | — | No |
| *atp*E | 137 | 0.081 | 0.500 | 0.500 | 1.02 (0.0%) | | 0 | 0 | 0 | — | No |
| *atp*F | 189 | 0.481 | 0.500 | 0.500 | 1.65 (0.0%) | | 0 | 0 | 0 | — | No |
| *atp*H | 81 | 0.000 | 0.500 | 0.500 | 5.10 (0.0%) | | 0 | 0 | 0 | — | No |
| *atp*I | 247 | 0.098 | 0.498 | 0.500 | 3.42 (1.7%) | | 0 | 0 | 0 | — | No |
| *ccs*A | 329 | 0.708 | 0.500 | 0.500 | 1.00 (13.7%) | | 0 | 0 | 0 | — | No |
| *cem*A | 229 | 0.340 | 0.500 | 0.500 | 8.97 (0.0%) | | 0 | 0 | 0 | — | No |
| *clp*P | 217 | 1.308 | 0.284 | 0.500 | 1.60 (73.7%) | | 0 | 0 | 0 | — | No |
| *inf*A | 77 | 1.316 | 0.351 | 0.500 | 1.74 (100.0%) | | 0 | 0 | 0 | — | No |
| *mat*K | 517 | 0.771 | 0.500 | 0.500 | 7.13 (0.0%) | | 0 | 0 | 0 | — | No |
| *ndh*A | 363 | 0.260 | 2.1e-05 | 3.1e-04 | >1000 (0.0%) | | 1 | 1 | 0 | — | **gene-wide/branch/site** |
| *ndh*B | 521 | >5 | 0.038 | 0.349 | 969.38 (100.0%) | | 0 | 0 | 0 | — | No |
| *ndh*C | 120 | 0.178 | 0.500 | 0.500 | 6.74 (0.0%) | | 0 | 0 | 0 | — | No |
| *ndh*D | 501 | 0.073 | 0.500 | 0.500 | 1.45 (1.5%) | | 0 | 0 | 0 | — | No |
| *ndh*E | 101 | 0.111 | 0.500 | 0.500 | 4.31 (0.0%) | | 0 | 0 | 0 | — | No |
| *ndh*F | 740 | 0.195 | 0.229 | 0.500 | 11.21 (0.9%) | | 0 | 1 | 0 | — | **site** |
| *ndh*G | 176 | 0.199 | 0.500 | 0.500 | 4.48 (0.0%) | | 0 | 0 | 0 | — | No |
| *ndh*H | 393 | 0.093 | 0.500 | 0.500 | 1.31 (1.1%) | | 0 | 0 | 0 | — | No |
| *ndh*I | 180 | 0.000 | 0.500 | 0.500 | 3.58 (0.0%) | | 0 | 0 | 0 | — | No |
| *ndh*J | 159 | 0.000 | 0.500 | 0.500 | 2.74 (0.0%) | | 0 | 0 | 0 | — | No |
| *ndh*K | 250 | 0.173 | 0.500 | 0.500 | 4.00 (0.0%) | | 0 | 0 | 0 | — | No |
| *pet*A | 320 | 0.343 | 0.500 | 0.500 | 1.06 (0.1%) | | 0 | 0 | 0 | — | No |
| *pet*B | 218 | 0.080 | 0.500 | 0.500 | 8.29 (0.0%) | | 0 | 0 | 0 | — | No |
| *pet*D | 164 | 0.190 | 5.5e-04 | 0.007 | >1000 (0.3%) | | 1 | 1 | 0 | P=0.0001 | **gene-wide/branch/site** |
| *pet*G | 37 | 0.000 | 0.500 | 0.500 | 4.99 (12.1%) | | 0 | 0 | 0 | — | No |
| *psa*A | 750 | 0.120 | 0.500 | 0.500 | 1.01 (0.0%) | | 0 | 0 | 0 | — | No |
| *psa*B | 734 | 0.040 | 0.253 | 0.500 | 26.15 (0.2%) | | 0 | 0 | 0 | — | No |
| *psa*C | 81 | 0.000 | 0.500 | 0.500 | 3.92 (0.0%) | | 0 | 0 | 0 | — | No |
| *psa*I | 36 | 0.000 | 0.500 | 0.500 | 5.18 (0.0%) | | 0 | 0 | 0 | — | No |
| *psa*J | 42 | 0.000 | 0.500 | 0.500 | 5.08 (0.0%) | | 0 | 0 | 0 | — | No |
| *psb*A | 353 | 0.059 | 0.500 | 0.500 | 1.00 (0.0%) | | 0 | 0 | 0 | — | No |
| *psb*B | 508 | 0.115 | 0.500 | 0.500 | 6.64 (0.0%) | | 0 | 0 | 0 | — | No |
| *psb*C | 473 | 0.083 | 0.500 | 0.500 | 1.33 (0.0%) | | 0 | 0 | 0 | — | No |
| *psb*D | 353 | 0.041 | 0.500 | 0.500 | 3.33 (0.6%) | | 0 | 0 | 0 | — | No |
| *psb*E | 83 | 0.000 | 0.500 | 0.500 | 3.82 (0.0%) | | 0 | 0 | 0 | — | No |
| *psb*F | 39 | 0.000 | 0.500 | 0.500 | 4.03 (12.5%) | | 0 | 0 | 0 | — | No |
| *psb*H | 73 | 0.367 | 0.500 | 0.500 | 7.60 (0.0%) | | 0 | 0 | 0 | — | No |
| *psb*I | 36 | 0.000 | 0.500 | 0.500 | 2.81 (0.0%) | | 0 | 0 | 0 | — | No |
| *psb*J | 40 | 0.973 | 0.500 | 0.500 | 2.59 (0.0%) | | 0 | 0 | 0 | — | No |
| *psb*K | 61 | 0.627 | 0.500 | 0.500 | 1.45 (17.7%) | | 0 | 0 | 0 | — | No |
| *psb*L | 38 | 0.000 | 0.500 | 0.500 | 5.55 (0.0%) | | 0 | 0 | 0 | — | No |
| *psb*M | 34 | 0.000 | 0.500 | 0.500 | 4.36 (0.0%) | | 0 | 0 | 0 | — | No |
| *psb*N | 43 | 0.259 | 0.500 | 0.500 | 1.00 (13.1%) | | 0 | 0 | 0 | — | No |
| *psb*T | 35 | 0.000 | 0.500 | 0.500 | 5.41 (0.0%) | | 0 | 0 | 0 | — | No |
| *rbc*L | 487 | 0.635 | 0.201 | 0.500 | 11.66 (1.2%) | | 1 | 2 | 1 | — | **branch/site** |
| *rpl*14 | 122 | 0.226 | 0.500 | 0.500 | 1.00 (0.0%) | | 0 | 0 | 0 | — | No |
| *rpl*16 | 141 | 0.403 | 0.500 | 0.500 | 1.01 (0.0%) | | 0 | 0 | 0 | — | No |
| *rpl*2 | 279 | 0.114 | 0.003 | 0.031 | >1000 (0.1%) | | 1 | 0 | 0 | P=4e-05 | **gene-wide/branch** |
| *rpl*20 | 148 | 1.054 | 1.5e-14 | 5.5e-13 | >1000 (3.0%) | | 6 | 4 | 1 | — | **gene-wide/branch/site** |
| *rpl*23 | 93 | >5 | 0.383 | 0.500 | 77.89 (96.5%) | | 0 | 0 | 0 | — | No |
| *rpl*32 | 57 | 0.125 | 0.500 | 0.500 | 2.22 (0.0%) | | 0 | 0 | 0 | — | No |
| *rpl*33 | 66 | 2.278 | 0.358 | 0.500 | 2.95 (75.1%) | | 0 | 0 | 0 | — | No |
| *rpl*36 | 37 | 0.214 | 0.068 | 0.500 | >1000 (1.2%) | | 0 | 0 | 0 | — | No |
| *rpo*A | 343 | 0.400 | 0.500 | 0.500 | 1.31 (0.0%) | | 0 | 0 | 0 | — | No |
| *rpo*B | 1075 | 0.197 | 0.500 | 0.500 | 3.09 (0.0%) | | 0 | 0 | 0 | — | No |
| *rpo*C2 | 1388 | 0.400 | 0.058 | 0.478 | 281.61 (0.0%) | | 0 | 1 | 0 | — | **site** |
| *rps*11 | 140 | 0.245 | 0.500 | 0.500 | 2.63 (0.0%) | | 0 | 0 | 0 | — | No |
| *rps*12 | 125 | 1.388 | 0.460 | 0.500 | 1.54 (100.0%) | | 0 | 0 | 0 | — | No |
| *rps*14 | 100 | 0.874 | 0.500 | 0.500 | 1.25 (0.0%) | | 0 | 0 | 0 | — | No |
| *rps*15 | 90 | 1.729 | 0.377 | 0.500 | 1.68 (100.0%) | | 0 | 0 | 0 | — | No |
| *rps*16 | 85 | 0.845 | 0.500 | 0.500 | 6.42 (1.9%) | | 0 | 0 | 0 | — | No |
| *rps*18 | 111 | 0.317 | 0.500 | 0.500 | 1.00 (0.0%) | | 0 | 0 | 0 | — | No |
| *rps*19 | 94 | 0.383 | 0.500 | 0.500 | 1.02 (0.0%) | | 0 | 0 | 0 | — | No |
| *rps*2 | 236 | 0.300 | 0.500 | 0.500 | 9.14 (0.0%) | | 0 | 0 | 0 | — | No |
| *rps*3 | 219 | 0.759 | 0.380 | 0.500 | 4.41 (15.3%) | | 0 | 0 | 0 | — | No |
| *rps*4 | 202 | 1.261 | 0.477 | 0.500 | 1.24 (100.0%) | | 0 | 0 | 0 | — | No |
| *rps*7 | 155 | 1.268 | 0.500 | 0.500 | | 8.83 (0.0%) | 0 | 0 | 0 | — | No |
| *rps*8 | 132 | 0.678 | 0.500 | 0.500 | | 2.96 (0.0%) | 0 | 0 | 0 | — | No |
| *ycf*1 | 1976 | 1.617 | <1e-16 | <1e-16 | | 284.97 (0.6%) | 7 | 33 | 1 | P<1e-16 | **gene-wide/branch/site** |
| *ycf*2 | 2330 | 2.974 | 3.6e-14 | 8.9e-13 | | 39.63 (7.4%) | 4 | 9 | 1 | — | **gene-wide/branch/site** |
| *ycf*3 | 171 | 0.134 | 0.110 | 0.500 | | >1000 (0.3%) | 0 | 0 | 0 | — | No |
| *ycf*4 | 184 | 0.120 | 0.500 | 0.500 | | 1.01 (0.0%) | 0 | 0 | 0 | — | No |
